# A Surgical Technique to Repair Perineal Body Disruption Secondary to Sexual Assault

**DOI:** 10.1055/s-0039-1695048

**Published:** 2020-04-28

**Authors:** Giulia Brisighelli, Marc A. Levitt, Richard J. Wood, Christopher J. Westgarth-Taylor

**Affiliations:** 1 Department of Paediatric Surgery, Chris Hani Baragwanath Academic Hospital, University of the Witwatersrand, Johannesburg, South Africa; 2 Department of Colorectal and Pelvic Reconstructive Surgery, Children’s National Hospital, Washington DC, United States; 3 Department of Pediatric Colorectal and Pelvic Reconstructive Surgery, Nationwide Children's Hospital, Columbus, Ohio, United States

**Keywords:** perineal injury, perineal trauma, sexual assault, perineal reconstruction, posterior sagittal anorectoplasty

## Abstract

Perineal trauma is uncommon in the pediatric population and it is estimated that 5 to 21% is secondary to sexual abuse. We aim to present a proposed surgical technique to repair perineal injuries secondary to sexual assault in female children. The technique is based on the posterior sagittal anorectoplasty (PSARP) for repairing anorectal malformations and, between 2017 and 2019, it was used to treat three girls (2 months, 2 years, and 8 years of age) with fourth-degree perineal injuries secondary to sexual assault. One of them underwent laparotomy and Hartmann's colostomy for an acute abdomen. Two underwent wound debridement and suturing and only had a stoma fashioned at 5 days and 6 weeks posttrauma, respectively. The perineal repair was performed 2, 6, and 7 weeks postinjury and done as follows: with the child prone in jack-knife position, stay-sutures are placed on the common wall between the rectum and the vagina. Using a needle tip diathermy, a transverse incision is performed below the sutures lifting the anterior rectal wall up. Stay sutures are then positioned on the posterior wall of the vaginal mucosa. The incision between the walls is deepened until the rectum and the vagina are completely separated. The deep and superficial perineal body is then reconstructed using absorbable sutures and an anterior anoplasty and an introitoplasty are performed. The stoma in each was closed 6 weeks postreconstruction. At follow-up, now 1 year or more postrepair, all patients have an excellent cosmetic outcome and are fully continent for stools.

## Introduction

The severity of perineal injuries in children can range from minor skin tears to severe lacerations involving the anal sphincter, urogenital tract, and can even involve intraperitoneal extension. 1 They can be due to blunt trauma, impalement injuries, obstetric injuries, or sexual abuse. The latter always needs to be suspected and, unfortunately, is a common cause of perineal injury in our setting. 2

There are controversies regarding the best way to assess and manage pediatric patients with perineal trauma with the vast majority of work focused on the acute management. For severe injuries, most authors recommend fashioning a colostomy with delayed perineal reconstruction. However, to our knowledge, no specific technique for perineal reconstruction has been described.

The aim of our study is to present three children with fourth-degree perineal injuries due to sexual assault and to describe a surgical technique derived from the posterior sagittal anorectoplasty (PSARP) approach, used for repairing congenital anorectal malformations to reconstruct the perineal body and sphincter complex with the goal of creating a good cosmetic result and to allow the patient to regain faecal continence.

After obtaining ethics-committee approval (M190292), the medical records of three patients who presented with perineal injuries secondary to sexual assault were retrospectively reviewed. Information regarding time of presentation, type of medical and surgical management, development of complications, and outcome in terms of bowel control was recorded.

An extensive literature review was conducted focusing on case reports, case series, and original articles involving the acute management of perineal injuries and perineal reconstruction in the pediatric population with special emphasis on articles focusing on perianal injuries due to sexual assault.

## Case Reports

### Case 1

**Fig. 1:**
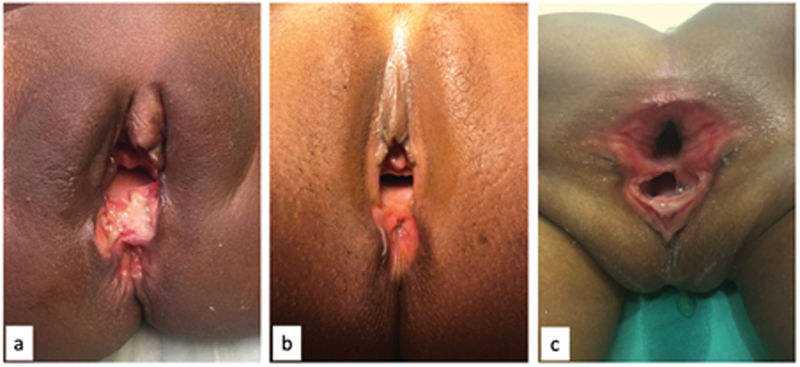
The three perineal injuries at examination under anesthesia (EUA) before the perineal repair was performed. ( **A** ) Case 1; ( **B** ) case 2; ( **C** ) case 3.

**Fig. 2:**
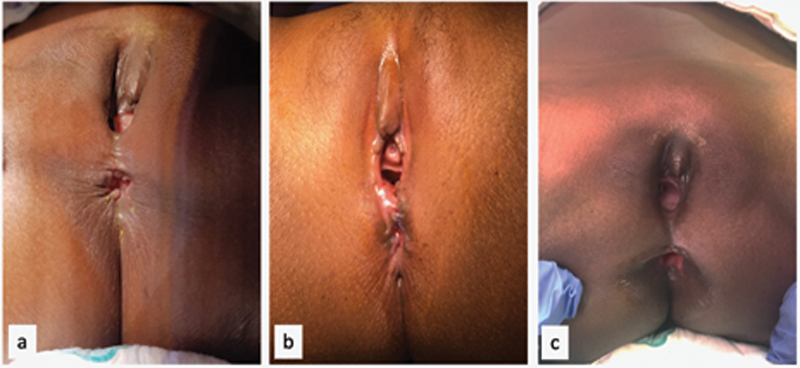
The three perineal injuries at EUA after the perineal repair was performed. ( **A** ) Case 1; ( **B** ) case 2; ( **C** ) case 3. EUA, examination under anesthesia.

A 2-month old girl with vaginal bleeding after sexual assault was brought to our Institution. She was immediately taken to the operating room for an examination under anesthesia (EUA). A tear of her vagina into her rectum, with partial involvement of her urethra, was observed ( Fig. 1A ). The wound was severely contaminated and was thus debrided and booked for a relook after 72 hours. At relook, a Hartmann's colostomy was fashioned. The perineal body was repaired 24 days postinjury, using a new surgical technique derived from the principles of the PSARP. Two months after the perineal reconstruction, an EUA and muscle sphincter stimulation revealed good anal tone with no evidence of a rectovaginal fistula, and her stoma was closed. She had an unremarkable postoperative recovery. At 2 years of age, she underwent another EUA that confirmed the correct location of the anus with an adequate perineal body, good sphincter contraction, a patent introitus, and the absence of a rectovaginal fistula ( Fig. 2A ). The patient is too young to be fully potty-trained, but she feels the urge to defecate and communicates it.

### Case 2

A 9-year old girl was brought to our institution after being found unconscious and naked in a field. Blood was noted around her genitalia and the decision was made to take her to the operating room for an EUA. A torn hymen, as well as a full thickness tear of the vagina, and disruption of the anal sphincter were identified. The surgeon on call attempted to approximate the perineal body without opening a stoma. An EUA performed 5 days later which revealed no signs of infection and good healing of the perineal body. After receiving all sexually transmitted disease (STD) and HIV prophylaxis, the child was discharged home to follow-up at the colorectal clinic. After being lost to follow-up, she presented 3 months postrepair with faecal soiling. It was determined that she had a total disruption of the perineal body and anterior sphincter complex ( Fig. 1B ). A repair of the perineal body using the new technique and a covering Hartmann's sigmoid stoma was performed. The stoma was reversed 45 days after the perineal reconstruction. Five months after the stoma closure, she was fully continent of stools ( Fig. 2B ).

### Case 3

A 2-year old girl presented to another institution with a vaginal injury involving the perineal body and rectum, and an acute abdomen after a sexual assault. She underwent an emergency exploratory laparotomy, an intraperitoneal vaginal laceration was identified and repaired, and a colostomy formed. Following this, she was referred to our center. She was taken to the operating room 45 days after the assault for an EUA ( Fig. 1C ). The rectum and vagina were contiguous, no perineal body was identified, the anal sphincter was completely absent anteriorly, and the dentate line was well preserved. A repair of the perineal body according to the new technique was performed. One month later, an EUA revealed an anus completely surrounded by sphincters, a patent introitus, and no rectovaginal fistula. The colostomy was closed 45 days after the reconstruction; 6 months later she was fully continent of stools ( Fig. 2C ).

### Medical Management

On arrival at the center, all the three children received all the protocolled STD prophylaxis, including HIV. At follow-up, they were tested for STD and HIV and referred to a psychiatrist for psychological support.

### Surgical Technique

**Fig. 3:**
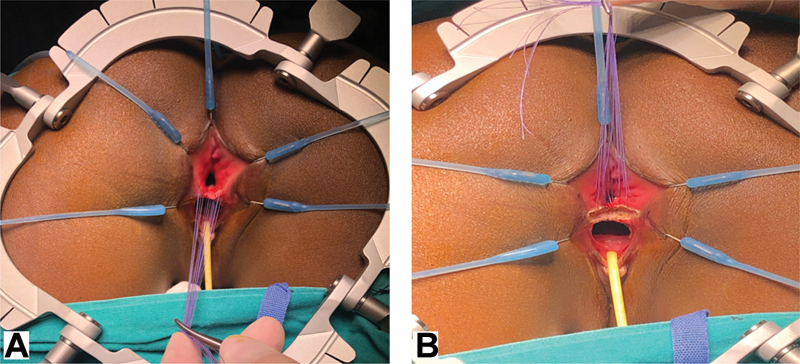
With the patient prone, a lone star retractor is positioned to facilitate exposure. The perineal reconstruction is then performed. ( **A** ) Stay sutures are positioned on the common wall between the rectum and the vagina. ( **B** ) Using a needle tip diathermy, an incision is made just below the stay sutures and the dissection begins to separate the anterior rectal wall from the posterior vagina.

**Fig. 4:**
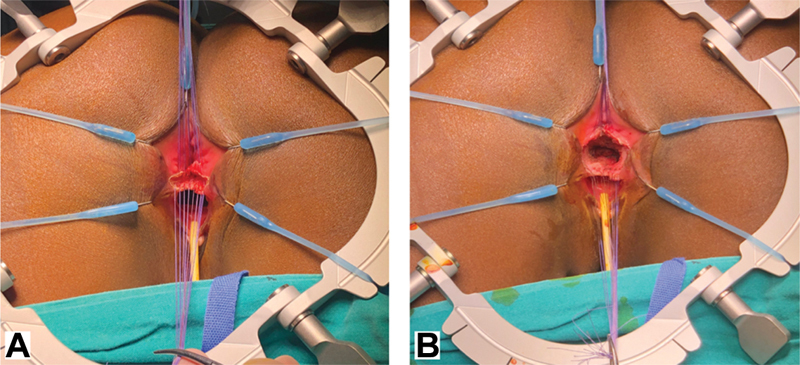
( **A** ) An additional line of stay sutures is then positioned on the vaginal mucosa. Exerting a countertraction on the two suture lines the dissection is continued to separate the rectum from the vagina. ( **B** ) The separation is only complete when an areolar plane has been reached between two structures.

**Fig. 5:**
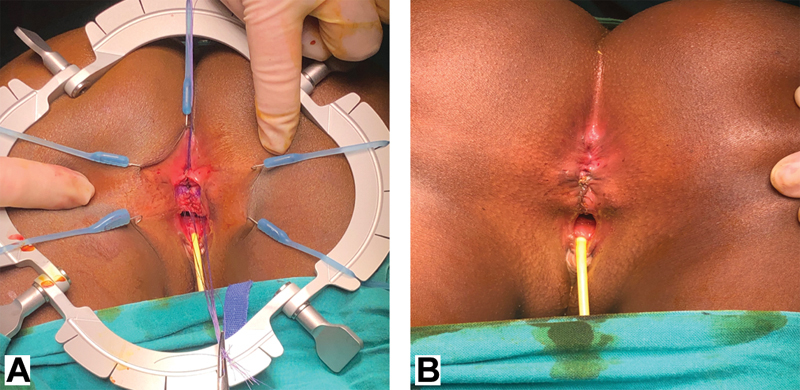
( **A** ) The perineal body is then reconstructed with interrupted absorbable sutures and the sphincter complex is reapproximated. ( **B** ) Reconstruction is completed with an anoplasty of the anterior rectal wall which is sutured to the reconstructed perineal body. The skin is then closed with interrupted absorbable sutures.

The surgical technique that was used followed principles of the PSARP technique to repair anorectal malformations (ARM). Figs. 3 – 5 illustrate the key steps of the approach.

A urinary catheter is placed and the patient is positioned prone with the hips elevated. Stay sutures are positioned on the common wall between the rectum and the vagina ( Fig. 3A ). Using a needle-tip diathermy, an incision is made just below the stay sutures and the dissection begins to separate the anterior rectal wall from the posterior vagina ( Fig. 3B ). A self-retaining retractor or a lone-star retractor can be used to provide exposure. An additional line of stay sutures is then positioned on the vaginal wall ( Fig. 4A ). Exerting a countertraction on the two suture lines, the dissection is continued to separate the two walls. The separation is only complete when the typical fibroareolar plane has been reached that exists between two structures ( Fig. 4B ). At this stage, the position of the sphincter complex is assessed with the muscle stimulator. The perineal body is then reconstructed with interrupted absorbable sutures and the sphincter complex is reapproximated ( Fig. 5A ). The key step involves bringing back together, anterior to the anus, the sphincter that was torn during the injury. If redundancy of the anal or vaginal mucosa is encountered, trimming may be appropriate. Reconstruction is completed with an anoplasty of the anterior rectal wall which is sutured to the now reconstructed perineal body. The skin is then closed with interrupted absorbable sutures ( Fig. 5B ).

Postoperatively antibiotics are given for 48 to 72 hours, the urinary catheter is kept in place for 24 hours and the patient can be discharged home after 48 hours if a covering colostomy is present. The stoma can be reversed once the perineal wound is healed and an EUA shows a patent anus completely surrounded by sphincter muscles.

## Discussion

Perineal trauma is uncommon in the pediatric population with a prevalence varying from 0.2 to 8% of all pediatric trauma. 3 4 According to the mechanism, injuries can be classified as blunt (77%) or penetrating (23%). 1 Falling from a height is the leading cause of perineal trauma (67% of cases), followed by motor vehicle collision (28%). 5 Obstetric injuries are also a possible cause of perineal trauma in the pediatric population but they are usually classified as a different entity and addressed separately in all the reports that we encountered. 6 7 It is estimated that 5 to 21% of pediatric perineal trauma is secondary to sexual abuse. 1 5 However, the true incidence and prevalence is not known and is likely underestimated, since most cases of sexual assault are under-reported by the victims because of the associated stigma. 2

**Fig. 6:**
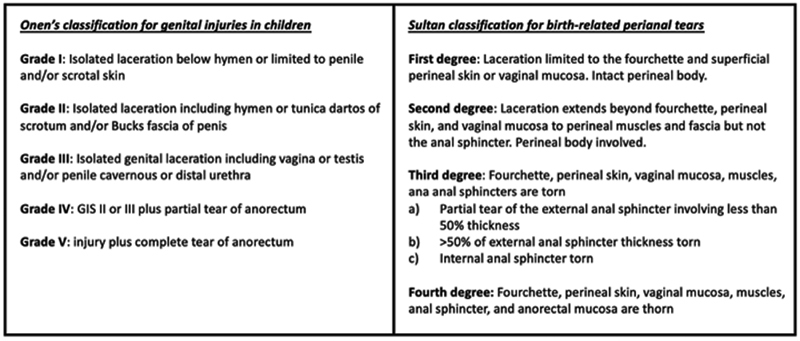
The two classifications used for perineal trauma. 1 8 GIS, genital injury score.

Perineal injuries can be classified according to the genital injury score (GIS) or according to the Sultan's classification ( Fig. 6 ). 1 8 The former is in an anatomical classification that can be used for children of both genders, and it groups perineal injuries into five different types according to the extent of the injury. 1 It does not take into consideration that the mechanism of the perineal injury, however, most of the injuries from sexual assault belong to 3rd and 4th degree. 1 The latter is specific for sexual assault injuries which is based on the adult classification of birth-related perineal tears in women and only applies to female patients. 8

All three cases have had with grade-4 injuries according to the GIS classification and with fourth-degree injuries according to Sultan's classification.

Most of the articles found on the topic focus on the acute medical and surgical management of patients with perineal injuries, with only rare case reports describing the specific technique used for the perineal reconstruction. 5 9 10

The pediatric patient who has experienced a sexual assault, the need for adequate resuscitation, collection of appropriate forensic evidence, investigation, treatment, and follow-up for STD and HIV is well documented. 11

General agreement exists to perform a physical examination under general anesthesia in all patients, at the time of presentation. Debridement of devitalized tissue and wound irrigation with saline and antiseptic solutions are considered the cornerstones of therapy. 9 The subsequent management depends on the extent of the injury and the degree of contamination. 1 5 9 While most authors agree that isolated genital injuries can be repaired primarily, controversy arises regarding the management of injuries with combined genital and anorectal involvement. 4 9 It has been demonstrated that the risk of postoperative complications due to primary perineal repair is higher in patients without a diverting colostomy, with more severe contamination, with prolonged delay before EUA, and with more severe genitoperineal injuries. 1 Therefore the safest approach consists of an initial wound disinfection and debridement, as well as a diverting colostomy, postponing the perineal repair to a later stage. 1 5 However, the role of routine faecal diversion has recently been questioned with more authors having attempted primary repair in patients with perineal trauma, especially if the injury is limited to the anus, without involving the rectum. 1 4 9

In our series, the only patient who underwent primary perineal repair without a covering stoma subsequently developed a wound dehiscence, with disruption of the anterior anal sphincter and soiling. Based on our experience, we believe that perineal injuries due to sexual assault are intrinsically associated with severe perineal contamination, because of the mechanism of trauma and the likelihood of delayed presentation, and should, therefore, all be treated with a diverting stoma and delayed perineal repair. 9 We believe that the repair can be safely performed 6 weeks after the injury, once the perineal wounds have healed, and the patients has completed treatments for STD and HIV.

Reconstructive surgery using muscle flaps and skin grafts has been described in patients with perineal trauma. 1 A Swenson's type endorectal pull-through has been performed in a child with pelvic disruption due to a gunshot wound and a posterior sagittal incision has been used to repair a rectovaginal fistula secondary to sexual assault. 4 10 Our technique is based on the principles learned from the PSARP. However, only the posterior vaginal wall and the anterior rectal wall are mobilized. The posterior aspect of the rectum is left completely untouched thus preserving nerves, sphincter muscles, and the anoderm. This approach allows excellent exposure without sacrificing nerves and muscles. Moreover, reaching the fibroareolar plane allows a complete separation between rectum and vagina and minimizes the risk of postoperative rectovaginal fistulae. The prone position is ideal as it provides the best visualization. We believe that this technique could also be used potentially for any acquired rectovaginal fistulae; HIV associated or trauma related. 12 In this case, the risk of recurrent fistulae can be minimized by the use of a fat pad to interpose between rectum and vagina. 13

The long-term results achieved both from cosmetic and functional points of view in our presented approach are very promising, with no surgically related complications observed so far. We are aware that the series in which we have used this new technique is too small to draw any significant conclusion; however, the outcomes are good in terms of bowel control and cosmesis.

## Conclusion

In our setting, perineal trauma due to sexual assault in children is unfortunately not a rare event. Patients may have genital injuries with anorectal involvement. At presentation, examination under anesthesia, wound irrigation, and local debridement should be performed in all children. Due to the intrinsic contamination of the wound and the likelihood of delayed presentation, a diverting stoma with postponed perineal repair should be the procedure of choice. We propose, this new surgical technique leads to successful outcome in terms of bowel control and good cosmetic results. The prone position and good repair of the perineal body, provided by separating fully the posterior vaginal wall from the anterior rectal wall, are the key elements.

## Notes

New Insights and the Importance for the Pediatric Surgeon Perineal trauma due to sexual assault in children may cause anorectal involvement and severe perineal body injuries. At presentation, examination under anesthesia, wound irrigation, and local debridement should be performed in all children. Due to the intrinsic contamination of the wound and the likelihood of delayed presentation, a diverting stoma with postponed perineal repair should be the procedure of choice. This new surgical technique for perineal reconstruction leads to successful outcome in terms of bowel control and good cosmetic results.
